# Coronary Artery Calcium Screening: Does it Perform Better than Other Cardiovascular Risk Stratification Tools?

**DOI:** 10.3390/ijms16036606

**Published:** 2015-03-23

**Authors:** Irfan Zeb, Matthew Budoff

**Affiliations:** 1 Department of Medicine, Bronx-Lebanon Hospital Center, 1650 Grand Concourse, Bronx, NY 10457, USA; 2 Department of Cardiology, Los Angeles Biomedical Research Institute at Harbor-UCLA Medical Center, Torrance, CA 90502, USA; E-Mail: mbudoff@labiomed.org

**Keywords:** coronary artery calcium, risk stratification, cardiovascular risk

## Abstract

Coronary artery calcium (CAC) has been advocated as one of the strongest cardiovascular risk prediction markers. It performs better across a wide range of Framingham risk categories (6%–10% and 10%–20% 10-year risk categories) and also helps in reclassifying the risk of these subjects into either higher or lower risk categories based on CAC scores. It also performs better among population subgroups where Framingham risk score does not perform well, especially young subjects, women, family history of premature coronary artery disease and ethnic differences in coronary risk. The absence of CAC is also associated with excellent prognosis, with 10-year event rate of 1%. Studies have also compared with other commonly used markers of cardiovascular disease risk such as Carotid intima-media thickness and highly sensitive C-reactive protein. CAC also performs better compared with carotid intima-media thickness and highly sensitive C-reactive protein in prediction of coronary heart disease and cardiovascular disease events. CAC scans are associated with relatively low radiation exposure (0.9–1.1 mSv) and provide information that can be used not only for risk stratification but also can be used to track the progression of atherosclerosis and the effects of statins.

## 1. Introduction

Atherosclerosis coronary artery disease is among the leading cause of morbidity and mortality in the Western world. The shear burden of cardiovascular disease on healthcare costs is enormous, with an estimate of 475 billion US dollars spent in the year 2009 alone [1]. By 2030, real total direct medical costs of cardiovascular disease (CVD) are projected to increase to ≈ $ 918 billion [2]. In order to drive the cost down, emphasis is on preventive measures and earlier detection of cardiovascular disease. There is a number of risk factors algorithms, biomarkers and imaging studies to screen for cardiovascular diseases. Framingham risk scores (FRS), Reynolds risk score, highly sensitive C-reactive protein (hs-CRP), carotid intima media thickness (CIMT) and coronary artery calcium (CAC) are among the various measures that can be used for screening of cardiovascular disease among asymptomatic population. CAC score has emerged as one of the strongest risk prediction tools. It represents calcific atherosclerosis in the coronary arteries and correlates well with the overall burden of atherosclerosis in the coronary arteries. The current review article will compare CAC score with the remaining risk stratification tools.

## 2. Framingham Risk Score and Coronary Artery Calcium

Framingham risk score is the most commonly used cardiovascular risk stratification tool in the general population due to its ease of use. It takes into account major cardiovascular risk factors including age, sex, dyslipidemia, smoking and hypertension [3]. This risk scoring system predicts an estimate of population risk for CVD events over 10-years and categorizes individual risk for developing CVD to low (10-year risk of <10%), intermediate (10-year risk of 10%–20%), and high (10-year risk of >20%) risk. FRS is easy to calculate and provides a good overall estimate of patient risk for future cardiovascular problems. However, there are several limitations to the use of this classification for risk assessment in the general population.

**Figure 1:**
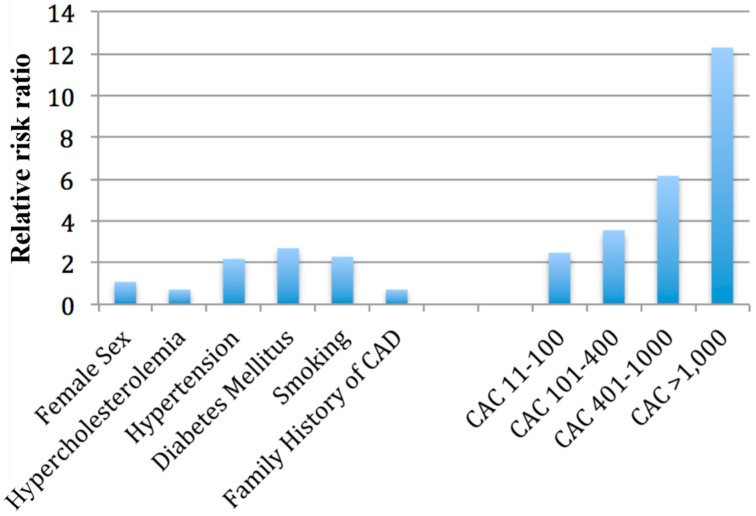
Predictive value of coronary artery calcium [4].

Shaw *et al.* [4] followed up a cohort of 10,377 asymptomatic individuals for mean duration of five years and compared cardiac risk factors such as family history of coronary artery disease, hypercholesterolemia, hypertension, smoking and diabetes mellitus with CAC score (Figure 1). CAC was found to be an independent predictor of mortality (*p* < 0.001) and it provided improved discrimination value compared to the cardiac risk factors alone (concordance index 0.78 *vs.* 0.72, *p* < 0.001) in a multivariable model for prediction of death. CAC was also superior to estimated Framingham risk score in outcome classification ability (Area under the ROC curve = 0.73 *vs.* 0.67, *p* < 0.001).

Greenland *et al.* [5] evaluated predictive value of CAC across increasing levels of FRS categories (0%–9%, 10%–15%, 16%–20% and ≥21%). For low risk FRS categories (0%–9%), CAC >300 did not provide an increased risk. CAC >100 was associated with increased risk among FRS categories 10%–15% and higher. The risk associated with FRS category 10%–15% and CAC score >300 (hazard ratio 17.6, *p* < 0.001) was comparable with the risk associated with FRS category >21% and CAC >300 (hazard ratio 19.1, *p* < 0.001). 2010 American College of Cardiology (ACC) and American Heart Association (AHA) guidelines [6] have incorporated CAC for cardiovascular risk assessment in asymptomatic adults at intermediate risk (10%–20% 10-year risk) (Class II a indication). The current clinical practice guidelines [6] do not recommend the use of CAC for low risk population risk assessment. Okwuosa *et al.* [7] evaluated the prevalence of CAC in low risk FRS categories and the number needed to screen to detect one individual with CAC score >300. There were 1.7% and 4.4% of the population found to have CAC score ≥300 within FRS categories of 0%–2.5% and 2.6%–5.0%, respectively. The respective number needed to screen were 59.7 and 22.7. The number needed to screen to detect persons with CAC score ≥300 decreased with increasing FRS categories; with only 4.2 and 3.3 individuals needed to be screened with FRS of 15.1%–20% and >20%. Okwuosa *et al.* [8] evaluated role of novel markers for CAC score progression among patients within low risk FRS category (<10%). They looked at the following variables: LDL particle number (LDLpn), urine albumin, CRP using a high-sensitivity assay, d-dimer, factor VIIIc, total homocysteine (tHcy), fibrinogen, cystatin C, soluble intercellular adhesion molecule-1 (sICAM-1) and carotid intima media thickness (CIMT). The study showed significant association of most of these risk factors with CAC progression in univariate and age adjusted models. This statistical significance was lost after adjustment for traditional cardiovascular risk factors. The study also evaluated predictive value of various combinations of novel markers with CAC progression compared with a base model composed of traditional risk factors. These combination models showed little or no improvement over the base model in discrimination and informativeness. Taylor *et al.* [9] performed a study evaluating the predictive value of CAC in 2000 healthy young men and women aged 40–50 years of age. A total of nine coronary events occurred, with four in men with 10-year FRS risk <6% and five men with 10-year FRS risk of 6%–10%. None of the events occurred in men with 10-year FRS risk above 10%. Seven out of nine men had CAC among those who suffered coronary heart disease (CHD) events. This study showed that the presence of any CAC was associated with an 11.8-fold increased risk of acute CHD (*p* = 0.002) after controlling for the FRS. 2010 ACC/AHA guidelines [6] suggest use of CAC for cardiovascular risk assessment of individuals with 10-year FRS risk of 6%–10% (Class II b indication). The current clinical practice guidelines do not recommend the use of CAC for low risk individuals (10-year FRS risk <6%) for cardiovascular risk assessment (Class III indication). The study performed by Greenland *et al.* [5] did not show any benefit of cardiovascular risk assessment with CAC score in the 10-year FRS category of less than 10%. The mean age of population at the time of computed tomography (CT) scan in this study was 65.7 years. The population groups in both Taylor *et al.* [9] (mean age 43 years) and Okwuosa *et al.* [7] were younger (mean age: 60.9 years, 53% women). It is already known that the FRS performs poorly in younger patients and women [10,11,12]. In a multi-ethnic study of atherosclerosis, 90% of the women were classified as low risk based on FRS risk classification [13]. There were 32% of low risk women who were found to have CAC score of more than 0 (four percent had CAC score ≥300). In these women, CAC score of >300 were highly predictive of future CHD and CVD events (6.7% and 8.6% absolute CHD and CVD risk, respectively) over a 3.75 year period. Ninety-five percent of the US women younger than 70 are found to be at low risk for coronary heart disease according to the data from Third National Health and Nutrition Examination Survey (NHANES III) [10] and, thereby, do not qualify for more aggressive medical management for standard risk factors according to the National Cholesterol Education Program Expert Panel on Detection, Evaluation, and Treatment of High Blood Cholesterol in Adults (Adult Treatment Panel III) (NCEP/ATP III) [14]. These persons otherwise may be classified as low-risk, and may not benefit from preventive treatments that can otherwise be offered to them based on CAC scores.

Elias-Smale *et al.* [15] evaluated the effect of CAC in risk assessment of elderly population (2028 subjects, age 69.6 ± 6.2 years and showed that CAC was able to reclassify 52% of men and women in intermediate risk category.

Family history of premature coronary artery disease represents a unique situation. The persons may suffer from cardiovascular events at an earlier age. The usual risk stratification tools such as FRS may perform poorly in these persons. Reynolds risk score includes parental family history of premature CAD and high-sensitivity C-reactive protein (hs-CRP) to traditional risk factors. CAC use has been assessed in this situation as well [16,17,18]. Nasir *et al.* [16] showed that the age-, gender- and race-adjusted prevalence of CAC >0 was significantly higher with presence of any family history of premature CHD than those with no family history of premature CHD among individuals classified as low risk (35% *vs.* 23%, *p* < 0.0001) and among those at intermediate risk (70% *vs.* 60%, *p* = 0.01). Similar results were seen with CAC ≥75th percentile among individuals at low risk (24% *vs.* 14%, *p* = 0.0003) and intermediate-risk (34% *vs.* 20%, *p* < 0.001) respectively. A *post hoc* analysis from St. Francis Heart study [17] revealed benefit of statin treatment in individuals with a family history of premature CAD and CAC above 80th percentile.

CAC has been shown to perform better across a wide range of FRS categories (6%–10% 10-year risk and 10%–20% 10-year risk). FRS is a population-based risk of 10-year event rates; however, individual persons may not be at the same level of risk in each category as determined by FRS. The individual risk can be further stratified with the use of various markers like hs-CRP and CAC. Polonski *et al.* [19] showed that addition of CAC to traditional risk factors improves risk prediction significantly compared to traditional risk factors alone. Traditional risk factors were able to classify 69% of the cohort into the highest or lowest risk categories, whereas addition of CAC to this model increased it to 77%. CAC along with traditional risk factors was able to reclassify 23% of those who had cardiac events into high risk and 13% without cardiac events into low risk.

Coronary atherosclerosis burden differs between different ethnic groups [20]. Framingham risk score does not account for the ethnicities. Detrano *et al.* [20] performed coronary calcium scans on population-based sample of 6722 men and women, of whom 38.6% were white, 27.6% were black, 21.9% were Hispanic, and 11.9% were Chinese. They found that adjusted risk for any coronary events for CAC score categories 1–100, 101–300 and >300 increased by a factor of 3.61-fold, 7.73-fold and 9.67-fold when compared with CAC score of zero (*p* < 0.001, respectively). The adjusted risk for any coronary events for ethnic groups Whites, Chinese, Black and Hispanics was 1.22-fold (*p* < 0.001), 1.36-fold (*p* < 0.005), 1.39-fold (*p* < 0.001) and 1.18-fold (*p* < 0.001), respectively. Across four ethnic groups, a doubling of CAC increased the risk of major coronary event by 15%–35% and the risk of any coronary event by 18%–39%. The areas under the receiver-operating-characteristic curve showed superior predictive value of CAC compared to the standard risk factors for both major and any coronary events.

The predictive value of increasing CAC score is well established. The protective value of CAC score zero is also very promising. A number of studies have consistently reported better outcomes associated with the absence of CAC (Table 1). These persons may require less intensive treatments and further diagnostic testing due to very low event rates in this population group. Blaha *et al.* [21] evaluated annualized all-cause mortality rates in 44,052 consecutive asymptomatic patients referred for CAC testing. They found that 45% had no CAC on screening and there were 104 deaths in those with CAC score zero. Patients with CAC score of 0 have an excellent prognosis, with a 10-year event rate of about 1%.

**Table 1:** Predictive value of zero coronary artery calcium score.

Author	Year	Total No. of Participants	Participants with CAC Score 0	Event Rate	Follow up Period
Blaha *et al.* [21]	2009	44,052	19,898	0.87/1000 person-years	Mean follow-up of 5.6 ± 2.6 years
Arad *et al.* [22]	2005	4613	1504	1/1000 person-years	4.3 years
Taylor *et al.* [9]	2005	2000	1263	0.6/1000 person-years	Mean follow-up of 3.0 ±1.4 years
Budoff *et al.* [23]	2007	25,253	11,046	0.6/1000 person-years	Mean follow-up of 6.8 ± 3 years
Detrano *et al.* [20]	2009	6722	3409	0.6/1000 person-years	3.7 years
Shaw *et al.* [4]	2003	10,377	5067	1.5 events/1000 person-years	Mean follow-up of 5.0 years
LaMonte *et al.* [24]	2005	10,746	2692	1.6 events/1000 person-years	3.5 years

The value of CAC in cardiac disease prediction has been well established. Herman *et al.* [25] evaluated association of CAC with stroke and showed that log10 (CAC + 1) is an independent predictor of stroke (hazards ratio, 1.52 (95% confidence interval, 1.19–1.92); *p* = 0.001) in addition to age, systolic blood pressure and smoking in subjects at low or intermediate vascular risk. This association was independent of atrial fibrillation detected at baseline and follow up exams in prediction of stroke (fully adjusted hazard ratio, 1.31 (1.00–1.71); *p* = 0.049) that may be the underlying cause for cryptogenic strokes. O’Neal *et al.* [26] showed that higher CAC scores were associated with increased risk for atrial fibrillation (CAC = 1–100: Hazard Ratio 1.4, 95% CI 1.01–2.0; CAC = 101–300: HR 1.6, 95% CI 1.1–2.4; CAC > 300: HR 2.1, 95% CI 1.4–2.9) in a model adjusted for sociodemographics, cardiovascular risk factors, and potential confounders.

The recently published ACC/AHA guidelines on the assessment of cardiovascular risk [27] used a different risk tool to estimate 10-year and long term risk of atherosclerotic cardiovascular disease (ASCVD) that incorporates age, sex, race, HDL-cholesterol, total cholesterol, diabetes, systolic blood pressure or treatment for hypertension and smoking status. This new risk stratification tool may replace FRS for risk prediction. However there are currently no studies available that compare CAC with 10-year ASCVD risk tool.

## 3. Reynolds Risk Score and Coronary Artery Calcium

Reynolds risk score (RRS) includes traditional risk factors used in FRS and adds parental family history of premature CAD and high-sensitivity C-reactive protein (hs-CRP) [28]. It provides improved prediction of CVD events in both men and women and has been proposed as an alternative to FRS in risk stratification [28,29]. DeFillepis *et al.* [30] compared the performance of FRS and RRS in predicting CAC incidence and progression among 5140 individuals in MESA population. Both FRS and RRS were predictive of incident CAC (relative risk: 1.40 and 1.41 per 5% increase in risk, respectively) and CAC progression (relative risk 6.92 and 6.82 per 5% increase in risk, respectively). There was discordance in risk category classification (<10% and >10% per 10-year HD risk) in 13.7% of patients. RRS was found superior to FRS in providing additional predictive information for incidence and progression of CAC when discordance between the scoring systems existed. Desai *et al.* [31] evaluated whether CAC leads to reclassification of RRS risk assessment in patients without prior CHD. There were 72% of patients with CAC score >400 and 88% of patients with high CAC percentile had low or intermediate risk by RRS risk classification. CAC can potentially improve risk reclassification for RRS risk assessment.

## 4. Carotid Intima Media Thickness and Coronary Artery Calcium

Carotid intima-media thickness (CIMT) represents carotid atherosclerosis and is being used as a surrogate marker for atherosclerosis. Its utility relies on its ability to predict future adverse cardiovascular events [32]. Lorenz *et al.* [33] performed a meta-analysis of eight studies where they showed that the age- and sex-adjusted overall estimates of the relative risk of 1.26 (95% CI 1.21–1.30) for myocardial infarction and1.32 (95% CI 1.27–1.38) for stroke for each one-standard deviation difference of common carotid IMT. CIMT does predict future CHD events but is a slightly better predictor for cerebrovascular events. Folsom *et al.* [34] compared CAC with CIMT in a cohort of 6698 subjects aged 45–84 years of age in four ethnic groups enrolled in MESA study and showed that the hazard of CVD increased 2.1 (95% CI 1.8–2.5) fold for log-transformed CAC *vs.* 1.3-fold (95% CI 1.1–1.4) for maximum CIMT for each standard deviation increment of log-transformed CAC and maximum CIMT. For coronary heart disease, the hazard ratios per 1-SD increment increased 2.5-fold (95% CI 2.1–3.1) for CAC score and 1.2-fold (95% CI 1.0–1.4) for IMT. The study also showed CAC to be a superior predictor of incident CVD compared with CIMT (area under the curve 0.81 *vs.* 0.78), respectively. Terry *et al.* [35] compared the performance of CAC and CIMT for non-invasive detection of prevalent CAD (≥50% stenosis in one or more coronary arteries identified on coronary angiography). CAC performed better than CIMT in terms of prevalent CAD for each quartile increase in both measures (odds ratio: 8-fold *vs*. 1.7-fold, respectively).

CAC seems to be better than CIMT in patients with atherosclerosis CAD. However, among patients with coronary micro-vascular dysfunction, CIMT seem to perform better than CAC score. Danad *et al.* [36] evaluated 120 patients without documented evidence of CAD to compare CAC with CIMT for prediction of minimal coronary vascular resistance, a measure used to detect coronary micro-vascular disease [37]. Using bivariate and multivariable regression analysis using backward elimination revealed that only CIMT was a predictor of minimal coronary vascular resistance. Naqvi *et al.* [38] showed that CIMT based mean vascular age (61.6 ± 11.4 years) was significantly higher than coronary calcium age (58.3 ± 11.1 years) (*p* = 0.001); both of these were significantly higher than mean chronological age (*p* < 0.0001 and *p* < 0.04, respectively). CIMT was able to upgrade or downgrade FRS by >5% compared with CAC (42% of CIMT cases *vs.* 17% of CAC cases).

Studies comparing CAC with CIMT in younger population groups, where the prevalence of CAC is very low, CIMT may perform better in those population groups. Davis *et al.* [39] performed a study in young population (33–42 years of age) evaluating the relationship of CAC with CIMT. The multivariate model showed low density cholesterol-C (*p* < 0.001), pack-year smoking (*p* < 0.005) and CAC (*p* < 0.05) were significant in men; and low density cholesterol-C (*p* < 0.001), systolic blood pressure (*p* < 0.01) and CAC (*p* < 0.05) were significant in women and were predictive of CIMT in this population.

Lester *et al.* [40] performed a study on relatively younger population, aged 36–59 years of age, comparing CAC and CIMT to detect subclinical atherosclerosis. A large proportion of population had a CAC score of zero (75%). There was evidence of carotid atherosclerosis in 47% of the population with CAC score of zero, showing the role of CIMT in low risk, relatively younger population group with CAC score of zero.

## 5. C-Reactive Protein and Coronary Artery Calcium

AHA/ACC recommends measurement of C-reactive protein (CRP) in men 50 years of age or older or women 60 years of age or older with low-density lipoprotein cholesterol less than 130 mg/dL can be useful in selection of patients for statin therapy [41]. These recommendations are based on JUPITER trial [42] assessing the benefit of statin treatment in apparently healthy persons with hyperlipidemia (LDL-C < 130 mg/dL) but with elevated high-sensitivity CRP levels (hs-CRP ≥ 2 mg/dL). Measurement of hs-CRP has been shown as an independent prediction of future vascular events and improves global classification of risk, regardless of the LDL cholesterol level [43,44,45,46,47,48,49]. Arad *et al.* [22] performed a study evaluating 4903 apparently healthy middle-aged persons comparing CAC to standard coronary disease risk factors and CRP that were followed for a period of 4.3 years. CAC was shown to perform better than CRP and to highly correlate with future CAD events in this study. Park *et al.* [50] performed a study evaluating 1461 subjects without coronary heart disease comparing CAC and CRP in predicting cardiovascular events in non-diabetic individuals. The study showed that both CAC was predictive of non-fatal myocardial infarction or coronary death and any cardiovascular events (*p* < 0.005) whereas CRP was only predictive of cardiovascular events (*p* = 0.03). Risk group analysis that are defined by increasing tertiles for CAC (<3.7, 3.7–142.1, >142.1) and the 75th percentile for CRP (>4.05 mg/L) were associated with increasing risk with increasing CAC and CRP. Relative risks for the medium-calcium/low-CRP risk group to high-calcium/high-CRP risk group ranged from 1.8 to 6.1 for MI/coronary death (*p* = 0.003) and 2.8–7.5 for any cardiovascular event (*p* < 0.001). Lakoski *et al.* [51] examined the MESA participants to determine how many individuals risk can be reclassified with the use of CAC and CRP. The study showed that 30% of the intermediate-risk subjects by FRS had CRP concentration greater than 3 mg/L and 33% had CAC scores more than 100 AU. In gender specific analysis, 49% of intermediate-risk women and 27% of intermediate-risk men had CRP >3 mg/L, compared with 33% of intermediate-risk men and women had CAC >100 AU. When gender specific cut points for CRP and CAC were use, the same percentage of intermediate risk women and men had CRP above 75th percentile (28% and 27%, respectively) whereas more intermediate-risk women 40%, CAC > 50) than men (25%, CAC > 180) had a high CAC score.

Blaha *et al.* [52] performed a study evaluating whether CAC score may further risk stratify a JUPITER-eligible population (LDL-C < 130 mg/dL and hs-CRP ≥ 2.0 mg/dL) in participants enrolled in MESA study. For CHD events, the five-year number needed to treat for CAC scores of 0, 1–100 and >100 was 549, 94 and 24, respectively. In JUPITOR trial, the five-year number needed to prevent the occurrence of one primary end point was 25 [42]. The presence of CAC was associated with 4.3-fold increased CHD (95% CI 2.0–9.3) and 2.6-fold increased CVD (95% CI 1.5–4.5), while hs-CRP was not associated with either CHD or CVD after multivariable adjustment [52].

Möhlenkamp *et al.* [53] performed a study involving 3966 subjects without known CAD or acute inflammation, followed for 5.1 ± 0.3 years, to determine whether combined presence of CAC and hs-CRP improves discrimination and stratification of coronary events and all-cause mortality. For coronary events, net reclassification improvement (NRI) was 23.8% (*p* = 0.0007) for CAC and 10.5% (*p* = 0.026) for hs-CRP. Addition of CAC to Framingham risk variables and hs-CRP improved discrimination of coronary risk but not *vice versa*. Among persons with CAC score of zero, hs-CRP >3 mg/L was associated with a significantly higher coronary event risk compared with hs-CRP <3.0 mg/L (*p* = 0.006). Reilly *et al.* [54] performed a study involving 914 participants from the Study of Inherited Risk of Coronary Atherosclerosis (SIRCA) who were free of clinical CAD and had a family history of premature CAD. The study showed that median CAC scores increased across ordinal CRP categories in women but not in men (Krushal-Wallis χ^2^ = 22.5, *p* < 0.001 in women, χ^2^ = 2.5, *p* < 0.29 in men, respectively). CRP levels were found to be significant predictors of CAC scores in women after adjusting for traditional risk factors, which was lost after adjustment for body mass index. There was no such association found between CRP and CAC in men.

## 6. Coronary Artery Calcium and Other Imaging Parameters for Risk Prediction

Various imaging markers that can be easily obtained from coronary artery calcium scan have been proposed for prediction of CHD and CVD events. Pericardial fat is present around the heart—encompassing the coronary arteries—has been shown to be a predictor of coronary events [55]. Budoff *et al.* [56] showed that thoracic aortic wall calcification to be a significant predictor of incident coronary events in women, independent of CAC. Zeb *et al.* [57] showed liver fat measured by CT scans to be an independent predictor of incident coronary heart disease events. Yeboah *et al.* [58] performed a study to assess whether addition of computed tomography risk markers thoracic aorta calcium (TAC), aortic valve calcification (AVC), mitral annular calcification (MAC), pericardial adipose tissue volume (PAT), and liver attenuation (LA) to FRS + CAC provide improved discrimination for incident CHD and incident CVD events. CAC, TAC, AVC and MAC were all significantly associated with incident CVD/CHD/mortality among intermediate risk subjects; CAC had the strongest association. The addition of CAC to the FRS provides superior discrimination especially in intermediate-risk individuals. Among participants with intermediate FRS risk, the addition of TAC, AVC, MAC, PAT, or LA to FRS + CAC resulted in a significant reduction in area of the curve for incident CHD (0.712 *vs.* 0.646, 0.655, 0.652, 0.648 and 0.569; all *p* < 0.01, respectively). The addition of CAC to FRS resulted in a superior discrimination for incident CHD in the intermediate-risk groups compared to when TAC, AVC, MAC, PAT and LA were added to FRS + CAC (0.024, 0.026, 0.019, 0.012 and 0.012, respectively). These risk markers are unlikely to be useful for improving cardiovascular risk prediction.

## 7. Conclusions

CAC score has been shown as the strongest predictor of incident coronary events and is able to reclassify low-to-intermediate risk groups and certain subgroups, especially women and young adults most of which may be classified as low risk by FRS risk stratification. Most of the adult population will have CAC scores of zero (Table 1). The prevalence of CAC scores increases with increasing age. There is a number of different cutoffs that are used to denote increased CHD risk [20,59], however, the most commonly used cutoffs of increased CHD risk are CAC score 1–100, CAC 101–300 and CAC > 300 [20]. Studies have shown that subjects who undergo CAC scans tend to have better compliance rates with medications compared with other groups, and promote a healthy behavior in these patients (a picture equals a thousand words) [60,61]. CAC scans can be used to evaluate the efficacy of treatment regimens over time. The accelerated progression of CAC is associated with adverse cardiovascular events [62,63,64,65]. There are certain concerns associated with the use of CAC scores. Among them, the foremost concern associated with CAC use is radiation exposure. The recent advances in imaging techniques has lowered the radiation exposure associated with CAC scans and is comparable to mammograms in terms in radiation exposure (0.9 to 1.1 mSv) [66,67]. The scan can be easily performed in an outpatient setting without any prior preparation. Medicare is currently reimbursing the cost of CAC scans for selected population groups. Another issue that can arise with the use of CAC scans are incidental findings which can drive the cost upward due to further downstream testing as a result of these incidental findings. The prevalence of incidental findings can range from 4% to 8% [68,69,70]. There is a number of cost effectiveness analysis studies that are looking at the cost associated with the use of CAC scans. However, the information obtained with CAC can be very useful for earlier detection, institution of statins and prevention of adverse cardiovascular events.
